# Electrically Conductive Polyetheretherketone Nanocomposite Filaments: From Production to Fused Deposition Modeling

**DOI:** 10.3390/polym10080925

**Published:** 2018-08-18

**Authors:** Jordana Gonçalves, Patrícia Lima, Beate Krause, Petra Pötschke, Ugo Lafont, José R. Gomes, Cristiano S. Abreu, Maria C. Paiva, José A. Covas

**Affiliations:** 1 Pólo de Inovação em Engenharia de Polímeros, University of Minho, 4500-058 Guimarães, Portugal; jordana.goncalves@piep.pt (J.G.); patricia.lima@piep.pt (P.L.); 2 Leibniz Institute of Polymer Research Dresden (IPF), Hohe Str. 6, 01069 Dresden, Germany; krause-beate@ipfdd.de (B.K.); poe@ipfdd.de (P.P.); 3 European Space Research and Technology Centre, Keplerlaan 1, NL-2200 AG Noordwijk, The Netherlands; ugo.lafont@esa.int; 4 CMEMS-UMinho, University of Minho, 4500-058 Guimarães, Portugal; jgomes@dem.uminho.pt (J.R.G.); csa@isep.ipp.pt (C.S.A.); 5 Institute for Polymers and Composites/i3N, University of Minho, 4800-058 Guimarães, Portugal

**Keywords:** PEEK, carbon nanotubes, graphite nanoplatelets, nanocomposites, filaments, fused deposition modeling (FDM)

## Abstract

The present work reports the production and characterization of polyetheretherketone (PEEK) nanocomposite filaments incorporating carbon nanotubes (CNT) and graphite nanoplates (GnP), electrically conductive and suitable for fused deposition modeling (FDM) processing. The nanocomposites were manufactured by melt mixing and those presenting electrical conductivity near 10 S/m were selected for the production of filaments for FDM. The extruded filaments were characterized for mechanical and thermal conductivity, polymer crystallinity, thermal relaxation, nanoparticle dispersion, thermoelectric effect, and coefficient of friction. They presented electrical conductivity in the range of 1.5 to 13.1 S/m, as well as good mechanical performance and higher thermal conductivity compared to PEEK. The addition of GnP improved the composites’ melt processability, maintained the electrical conductivity at target level, and reduced the coefficient of friction by up to 60%. Finally, three-dimensional (3D) printed test specimens were produced, showing a Young’s modulus and ultimate tensile strength comparable to those of the filaments, but a lower strain at break and electrical conductivity. This was attributed to the presence of large voids in the part, revealing the need for 3D printing parameter optimization. Finally, filament production was up-scaled to kilogram scale maintaining the properties of the research-scale filaments.

## 1. Introduction

Additive manufacturing (AM) techniques allow the production of geometrically complex parts from three-dimensional (3D) model data, usually layer upon layer, without the need for additional tools or molds [1,2]. AM gradually evolved from a niche method for rapid prototyping to a competitive manufacturing process. The technique is expected to achieve substantial societal impact on various sectors such as healthcare, transportation, aerospace, electronics, construction [3], improved manufacturing sustainability, and simplified supply chain to increase efficiency in demand satisfaction [4].

Despite the diversity of AM techniques [3,5,6], only a few seem to meet the practical requirements of industrial manufacturing of small series. Fused deposition modeling (FDM) is one of these [7]. It was patented by S. Crump in 1989 [8] and involves pushing a plastic rod through a heated nozzle and depositing the molten extruded thin filament onto the platform as a vertical series of horizontal two-dimensional (2D) slices of the 3D part being manufactured. Compared to other AM techniques, FDM involves lower costs, is more user friendly, requires less post-processing (for example, microwave treatment to improve layer adhesion), and can use a multitude of materials. Nevertheless, the variety of commercially available materials is narrow—it basically includes acrylonitrile butadiene styrene (ABS) and polylactic acid (PLA) (the two most popular), polyethylene terephthalate (PET), nylon, thermoplastic polyurethane (TPU), poly(methyl methacrylate) (PMMA), polypropylene (PP), and polycarbonate (PC) [9,10], especially when compared to the range offered for well-established processes like injection molding or extrusion [7]. Consequently, one of the current major challenges of FDM is to increase the available palette of materials. The latter should yield improvements in the ease of printing, e.g., strength of the adhesion between filaments, deposition speed, dimensional accuracy, mechanical performance (see a recent review in Reference [6]), service temperature, and in generating specific functionalities (e.g., shape memory effects [11]), as well as thermal and/or electrical conductivity.

Polymer-based composites and nanocomposites seem particularly suitable for this purpose, and are, thus, the focus of substantial research efforts (see, for example, a recent review in Reference [10]). ABS filled with organically modified montmorillonite improved the mechanical performance of printed parts [12], while the addition of multi-walled carbon nanotubes (MWCNT) from 1 to 8 wt % increased stiffness and yield properties, and reduced the strain at break. Also, the electrical resistivity of the composite was strongly decreased by the addition of CNT; however, 3D printing led to a partial deterioration of this behavior [13]. ABS/graphene composites were 3D printed for the first time in 2015 [14]. The addition of 4 wt % graphite nanoplates (GnP) increased the elastic modulus and the thermal stability of 3D-printed parts, but decreased both stress and strain at break [15]. In the case of PLA, the addition of 5 wt % CNT increased the Young’s modulus of the FDM parts by 30%, but the tensile strength and toughness decreased 11% and 22%, respectively [16]. The incorporation of 10 wt % GnP reportedly improved the mechanical and thermomechanical properties, while the dielectric constant became quasi-independent of frequency [17]. Adding carbon fillers to polybutylene terephthalate (PBT) and polyamide 12 (PA12) filaments led to better mechanical and transport properties [18,19].

Recently, research was also directed toward the use of high-temperature engineering polymers in FDM. Polyetheretherketone (PEEK) combines excellent mechanical properties, good chemical resistance, and a high glass-transition temperature. Experimental evidence showed that, provided the operating conditions are set adequately, PEEK is quite suitable for FDM (articles [20,21,22] and references therein), and compares favorably with other materials such as PC and ABS [23,24]. For example, very recently, PEEK was successfully used to print a custom-designed rib prosthesis, and the mechanical behavior was found to be close to that of a natural rib [25]. The incorporation of 30% short carbon fibers into a PEEK matrix was recently proposed to strengthen the ribs of a space membrane structure [26]. Berretta et al. [22] printed PEEK/CNT nanocomposites with 1% and 5% CNT. Although the presence of CNTs did not seem to influence the mechanical performance of the parts produced with PEEK alone, every step of processing the composites (i.e., compounded composite feedstock filaments, single FDM-deposited layers, and fabricated test specimens) originated structures of lower performance.

The objective of the present work was to develop electrically conductive PEEK-based filaments (conductivity values above 10^−2^ S/cm) with a nominal diameter of 1.75 mm, suitable for FDM, exhibiting good mechanical properties, and obtained by melt compounding and plasticating extrusion methods scalable to industrial production (and at the kilogram scale). The research carried out on polymer nanocomposites containing carbon nanoparticles showed that lower percolation concentrations and higher electrical conductivity levels are typically achieved in composites with CNT compared to those with GnP. Moreover, it was also reported that synergistic effects in hybrid/ternary polymer composites filled with CNT and GNP cause a significant decrease in the percolation threshold in comparison with the binary equivalents, and that the effect on transport properties of combining different carbon particles is higher than the sum of the effects of the individual fillers [27,28,29,30,31,32]. It was suggested that a morphology consisting of CNTs placed between the GnP and forming bridges implies a less effective electrical network, but a better combination of properties was consistently reported [33].

Therefore, in the present work, hybrid/ternary nanocomposites of PEEK/CNT/GnP with different compositions were prepared by melt mixing using a co-rotating twin-screw extruder, and their electrical conductivity was measured. Selected nanocomposites were then extruded into filaments, which were also characterized. Finally, selected filaments were utilized to manufacture tensile bars using a commercial 3D printer. Although every step of processing produced composites with lower electrical conductivity, values of the order of 10 S/m were attained in the filaments produced. Due to this successful outcome, the European Research Agency tested these filaments in their satellites program [34].

## 2. Materials and Methods

### 2.1. Materials

PEEK Victrex 450 G^®^, with a Newtonian plateau melt viscosity of 350 Pa·s (400 °C) and a density of 1.30 g/cm^3^ was obtained from Victrex (Lancashire, UK). This particular grade was developed for applications for higher strength and stiffness as well as high ductility, and is suitable for sterilization for medical and food contact applications. The commercial carbon nanoparticles selected are identified in Table 1, which also presents their morphological and physical characteristics according to the manufacturer.

### 2.2. Experimental Design

**Figure 1:**
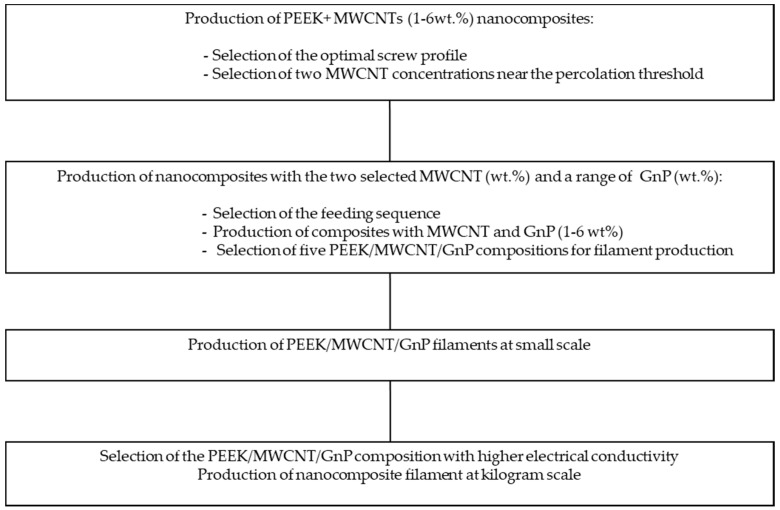
Main steps for nanocomposite optimization and the selection of filament composition, for the production of electrically conductive filaments for three-dimensional (3D) printing. PEEK—polyetheretherketone; MWCNT—multi-walled carbon nanotubes; GnP—graphene nanoplayes.

The sequence of processing steps taken to define the best processing conditions and hybrid composition of the target composite filament is presented in Figure 1. The selection was based on the electrical properties of the composites produced, a key property for the desired filament to be produced for 3D printing.

### 2.3. Processing

**Figure 2:**
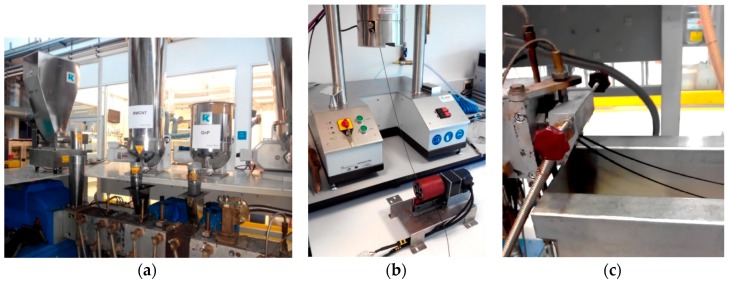
Manufacture of PEEK/CNT/GnP nanocomposites by melt compounding in a twin-screw extruder using separate feeding (**a**), and extrusion of the filaments using a capillary rheometer (**b**) or a twin-screw extruder (**c**).

The nanocomposites were manufactured and pelletized using a Coperion ZSK 26 (*L*/*D* = 40, Coperion GmbH, Stuttgart, Germany) co-rotating intermeshing twin-screw extruder and downstream accessories (water cooling bath, counter-current air dryer, and rotating knife). Various screw profiles were tested, each with a different number and geometry of conveying, kneading, and distributive mixing elements, in order to create diverse magnitudes of the thermomechanical stresses. The screw configuration that enabled the production of materials with higher electrical conductivity was selected. It contained four mixing zones separated by conveying elements. The mixing zone upstream consisted of a sequence of staggered kneading disks followed by a left-hand element, with the aim of melting the polymer; the second mixing zone comprised four sequential kneading blocks with different staggering angles, in order to induce dispersive mixing; the last two mixing zones downstream encompassed a combination of kneading blocks and toothed mixing elements, in order to guarantee both dispersive and distributive mixing. Joint as opposed to separate feeding of the components yielded similar results. The various nanocomposites were then prepared using a total feed rate of 3 kg/h, a screw speed of 300 rpm, and a set temperature profile increasing from 360 upstream to 375 °C at the die. PEEK was fed first and forced to melt, then MWCNT was added, followed by GnP (Figure 2a).

The composites produced were pelletized and filaments were obtained following two routes: (i) 10-m-long filaments with a diameter of 1.75 ± 0.03 mm were extruded using a Göttfert Rheo-Tester 2000 capillary rheometer (GOETTFERT Werkstoff-Prüfmaschinen GmbH, Buchen, Germany) set to 360 °C and developing an average shear rate at the die of approximately 110 s^−1^, coupled with a pulling unit operated under controlled speed (Figure 2b); (ii) spools with filaments with a diameter of 1.75 ± 0.04 mm were produced using the same Coperion extruder and operating conditions selected for the manufacture of the nanocomposites (Figure 2c), controlling the diameter with a set of two pulling rolls with independently controlled speeds sequentially positioned along the extrusion line, followed by automatic winding. The former is a batch process using approximately 100 g, which was adopted to produce filaments with a wide range of compositions, while the latter is an industrial process that was employed to demonstrate the production of filaments at the kilogram scale. The 3D-printed tensile test specimens were manufactured on an INDMATEC HPP 155 3D printer (Indmatec, Karlsrhue, Germany) using the 1.75-mm-diameter PEEK/MWCNT/GnP filaments. The printing conditions were as follows: an extrusion temperature of 400 °C, a build plate temperature of 100 °C, a layer height of 0.1 mm, an infill of 100%, a raster layer orientation of −45°/+45°, and a printing speed of 20 mm/s. Only one outline/perimeter shell was used in this printing strategy. The specimens were produced with a dumbbell shape printed in the XY build direction following the ASTM D638 type V standard. The slicing process to generate the G-code was done using the “Simplify 3D” software, (3.0, Simplify3D, www.simplify3d.com).

### 2.4. Characterization

#### 2.4.1. Nanocomposites

Melt flow index (MFI) measurements were performed based on the ISO 1238 standard, using the MFI GOTTFERT MI-3 equipment (GOETTFERT Werkstoff-Prüfmaschinen GmbH, Buchen, Germany) at 360 °C and a 10-kg load.

The electrical conductivity was measured on the extrudates using an LCR Quadtech 1920 equipment (Chroma Systems Solutions, Inc., Foothill Ranch, CA, USA), performing the measurements under direct current (DC). A two-contact-point configuration was adopted, with a distance of 60 mm between the measuring electrodes. A simple demonstrator consisting of a series of five 1.14-W light-emitting diodes (LEDs) was set up to illustrate the filament conductivity.

#### 2.4.2. Nanocomposite Filaments

The electrical conductivity of the filaments was measured as described above.

The filament diameter was measured in line every minute using a digital caliper. The filaments were observed with a Leica Digital Microscope DVM6 (Leica Microsystems Inc., Buffalo Grove, IL, USA). The filament cross-sections, obtained by cryo-fracture, were observed by scanning electron microscopy (SEM) performed with a NanoSEM FEI Nova 200 microscope (ThermoFischer Scientific, Hillsboro, OR, USA).

The filaments were tensile tested following the ASTM D2256 standard using a Shimadzu AG-X equipment (Shimadzu Corporation, Kyoto, Japan) fitted with pneumatic gripping jaws and a 1-kN load cell. A gauge length of 100 mm and a crosshead speed of 50 mm/min were used.

Differential scanning calorimetry (DSC) measurements were performed on a TA Instruments Q 20 equipment (TA Instruments, New Castle, DE, USA). Each sample was heated from 100 to 400 °C at a heating rate of 10 °C/min under nitrogen atmosphere, cooled at 10 °C/min to 100 °C, and heated again from 100 to 400 °C at 10 °C/min. The results reported are the average of two samples tested.

The measurements of thermal conductivity and thermoelectric properties were carried out on compression-molded plaques with 12.5-mm diameter and 2-mm thickness, obtained by melting the filaments at 370 °C for 1 min at 100 kN using the hot press PW40EH (Paul-Otto Weber GmbH, Remshalden, Germany). Thermal conductivity was measured at 25 °C on the light flash apparatus LFA 447 NanoFlash (Netzsch-Gerätebau GmbH, Selb, Germany). The thermoelectric (TE) properties were measured with a self-made set-up developed at IPF Dresden [35] at a temperature of 40 °C. The samples were painted with conductive silver at the ends, mounted on two copper blocks, and fastened with mounting clamps to ensure good contact. For all measurements, one block was kept at 40 °C, while the other was heated up in a controlled manner by carrying out temperature variations from 33 to 47 °C in steps of 2 K. The distance between the two blocks was set to 12 mm. The generated thermoelectric voltage ∆*V* was measured by a Keithley Multimeter 2001 (Keithley Instruments, Solon, OH, USA). The temperature of the two blocks was continuously monitored with K-type thermocouples to determine the temperature gradient (∆*T*). The Seebeck coefficient (*S*) was derived from the slope of ∆*V* vs. ∆*T* curves by linear fitting. In addition, the electrical resistivity (four-point measurement) was measured in the same device using a Keithley Multimeter 2001 (Keithley Instruments, Solon, OH, USA) between the two copper blocks after setting both to the same temperature. The thermoelectric performance of the material was evaluated by a dimensionless figure of merit (ZT) which is calculated by [36]

(1) $$ZT=\frac{\sigma^{2}ST}{\kappa },$$

where *σ* is the electrical conductivity, *S* the Seebeck coefficient, *T* the temperature, and *κ* the thermal conductivity. The term *σ^2^S* represents the power factor (PF). The results are based on mean values of three measurements.

The tribological characterization was carried out in a Bruker tribometer (model UMT-2, Billerica MA, USA), using a ball-on-flat reciprocating sliding configuration. Stainless-steel balls (5-mm diameter) were made to slide, without lubrication, against the filaments under a constant load of 1 N and oscillating frequency of 1 Hz.

#### 2.4.3. 3D-Printed Specimens

The dumbbell specimens produced by 3D printing were tensile tested on a Zwick/Roell Z100 (Zwick GmbH & Co. KG, Ulm, Germany) using a 2.5-kN load cell and a cross-head speed of 1 mm/min. The cross-sections of the tensile specimens were cut into 15-μm-thickness samples using a microtome, and were observed under an optical microscope, Olympus BH2, in transmission mode.

The electrical characterization of the tensile specimens was performed by depositing silver electrodes on the specimen surface and measuring the volume electrical conductivity (through the thickness) by varying the potential from −10 to 10 V, measuring the corresponding current intensity on a Keithley 487 picoammeter (Keithley Instruments, Solon, OH, USA).

## 3. Results and Discussion

### 3.1. Nanocomposites

**Figure 3:**
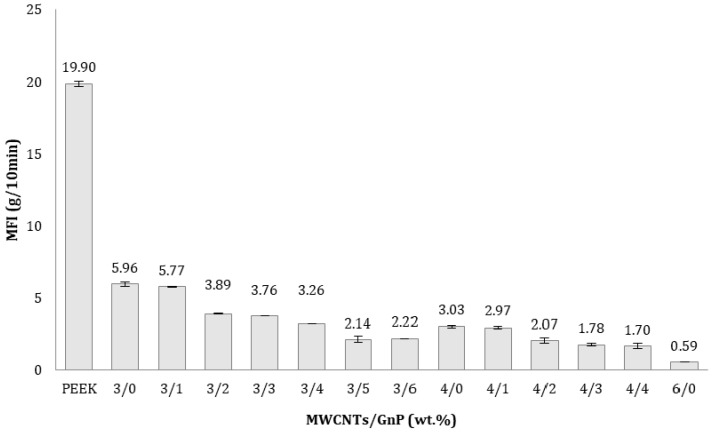
Effect of incorporating carbon nanoparticles on the melt flow index (MFI) of PEEK nanocomposites with different compositions.

Table 2 presents the effect of reinforcing PEEK with carbon fillers on representative twin-screw processing parameters, namely torque percentage consumed by the motor, pressure drop, and melt temperature at the die inlet. It shows that increasing CNT content caused a gradual increase in these parameters. For a die temperature set to 375 °C, a melt temperature of 368 °C was measured when processing PEEK, while when processing the MWCNT composites with incrementing loadings, it increased steadily up to 386 °C. These trends are the consequence of a gradual increase in viscosity that boosts viscous dissipation. When GnP was incorporated (at a constant content of MWCNT of 3 wt %) fluctuations of torque and pressure were observed, but the increase in pressure drop and melt temperature was reduced. For example, the addition of 3 wt % CNT to PEEK increased melt pressure and temperature from 21–24 bar and 368 °C to 28 bar and 376 °C, respectively, but a further addition of 3 wt % GNP (to attain a total of 6 wt % carbon fillers) induced a marginal raise to 23–30 bar and 379 °C, respectively. These last two values could also be directly confronted with 37 bar and 386 °C obtained for the composite with 6 wt % MWCNT. Thus, adding GnP to PEEK has a less adverse effect upon processability as compared to MWCNT. This can also be demonstrated through the melt flow index (MFI), a simple rheological index well accepted by industry. As seen in Figure 3, the presence of carbon nanoparticles significantly reduced the MFI. More specifically, adding 3 wt % CNT to PEEK reduced the MFI from 19.90 to 5.96 g/10 min, i.e., more than three fold. Increasing that percentage to 4%, MFI further dropped by roughly half, reaching 3.03 g/10 min. However, the addition of 1 GnP to 3 wt % CNT, yielding a similar overall nanoparticle content of 4 wt %, caused a marginal MFI reduction from 5.96 to 5.77 g/min.

**Figure 4:**
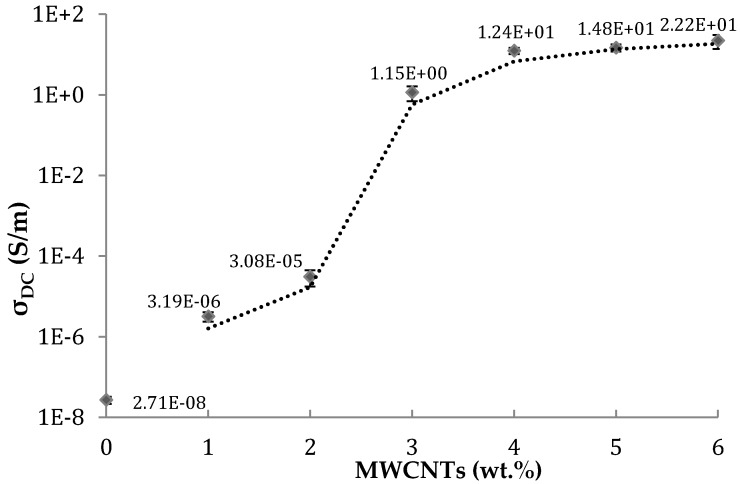
Direct current (DC) electrical conductivity of PEEK/MWCNT nanocomposites as a function of MWCNT content.

**Figure 5:**
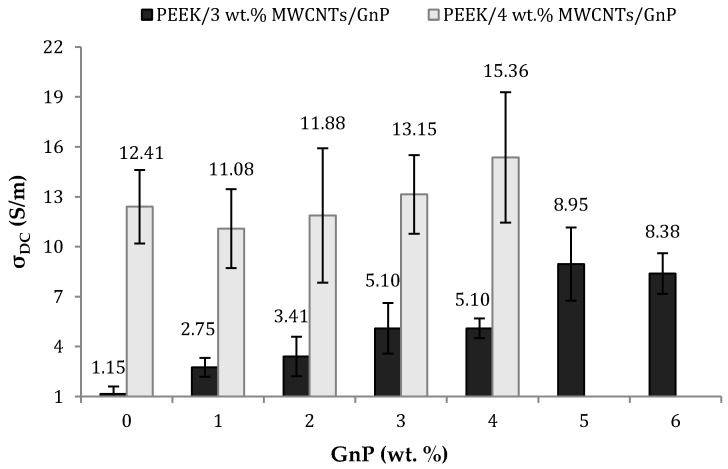
DC volume electrical conductivity of PEEK/MWCNT/GnP nanocomposites as a function of GnP content (at CNT contents of 3 and 4 wt %).

Figure 4 depicts the variation of the DC volume electrical conductivity with the concentration of MWCNT for PEEK/MWCNT nanocomposites. The maximum practical CNT loading was 6 wt %, as, beyond that value, the extrudate showed rheological anomalies. The percolation threshold occurred between 2 and 3 wt % CNT, which is within the typical range for nanocomposites prepared by melt compounding [37,38,39]. The electrical conductivity attained at higher concentrations (10–20 S/m) compare quite favorably with the results reported in the literature for composites with different matrices, the highest values reaching 1 S/m for a PP/4 wt % CNT system [39] and 6–7 S/m for a PC/2.5 wt % CNT composite [40]. Since a major aim of the work was to produce highly conductive filaments, the MWCNT concentrations of 3 and 4 wt % were selected to carry out the study of the effect of GnP addition on the composite properties. The effect of adding GnP to PEEK/CNT nanocomposites containing 3% or 4% CNT is displayed in Figure 5. An increase in the loading of GnP induces a growth, albeit moderate, of the electrical conductivity of the hybrid nanocomposites. Combinations of 3 wt % CNT with higher loads of GnP, or of 4 wt % CNT with lower loads of GnP, show a consistent electrical conductivity near or above 10 S/m.

**Figure 6:**
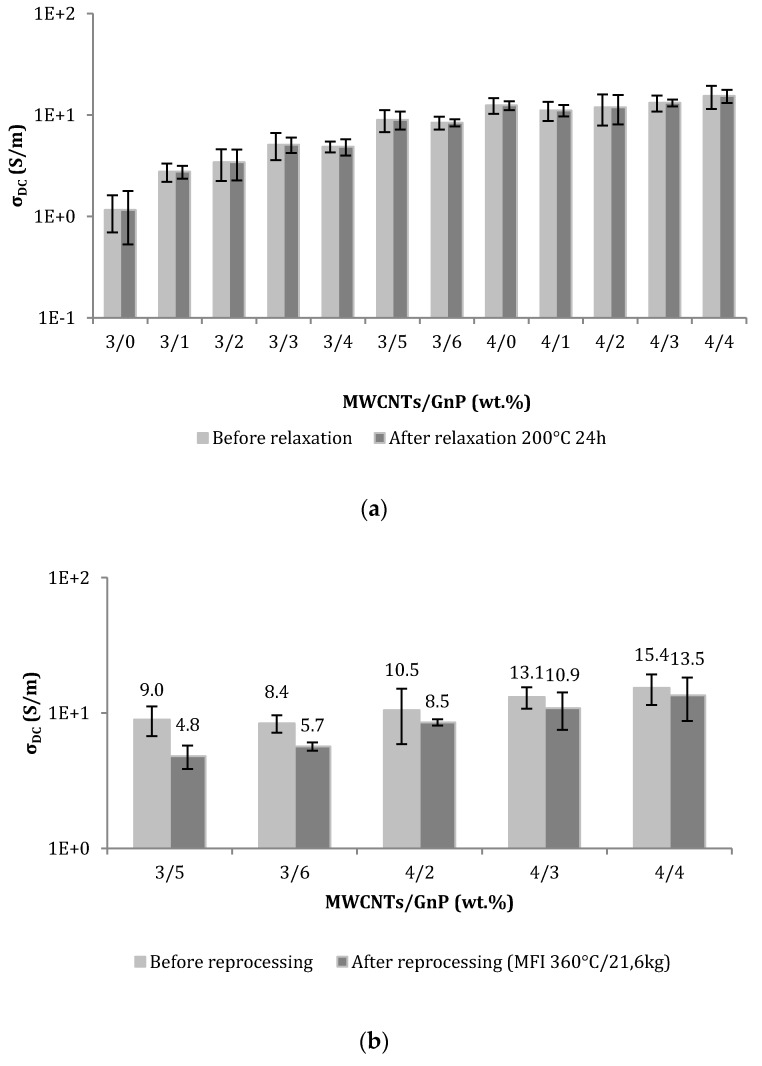
DC volume electrical conductivity of PEEK/MWCNT/GnP nanocomposites before and after a second heating/flow cycle: (**a**) annealing at 200 °C; (**b**) reprocessing at 360 °C.

Materials for FDM applications should also present good morphology stability since they will be successively subjected to compounding, filament extrusion, and 3D printing, all stages involving heating above the polymer melt temperature and flow. Evidence of re-agglomeration during re-heating and flow of polymer/CNT and polymer/GnP nanocomposites was well documented, as well as its influence on the resulting electrical performance [41,42,43,44]. The nanocomposites’ morphological stability upon heating was estimated by submitting the nanocomposite filaments to additional thermomechanical cycle(s) to observe eventual variations in the properties. The data shown in Figure 6 illustrate the effect of a second heating (annealing) and heating/flow cycle on the electrical conductivity of several PEEK/CNT/GnP nanocomposites prepared in this study. When the nanocomposites were re-heated and kept at 200 °C for 24 h (i.e., above *T*_g_, which is approximately 150 °C according to the manufacturer) (Figure 6a) no alterations in electrical conductivity were measured. Reprocessing of the nanocomposites at 360 °C using an MFI tester fitted with a standard weight of 21.6 kg (Figure 6b) induced a decrease in conductivity between 13% and 47%, depending on composition. Therefore, a progressive decrease in the electrical conductivity from composite to filament and to printed part is anticipated. This effect was previously observed, but not discussed, for PEEK/CNT composites [22].

Based on the results obtained for processability and electrical conductivity, the following compositions were selected for the production of composite filaments, expressed in MWCNT wt. %/GnP wt %: 3/1, 3/3, 3/5, 4/1, and 4/3.

### 3.2. Filaments

#### 3.2.1. Filament Morphology

**Figure 7:**
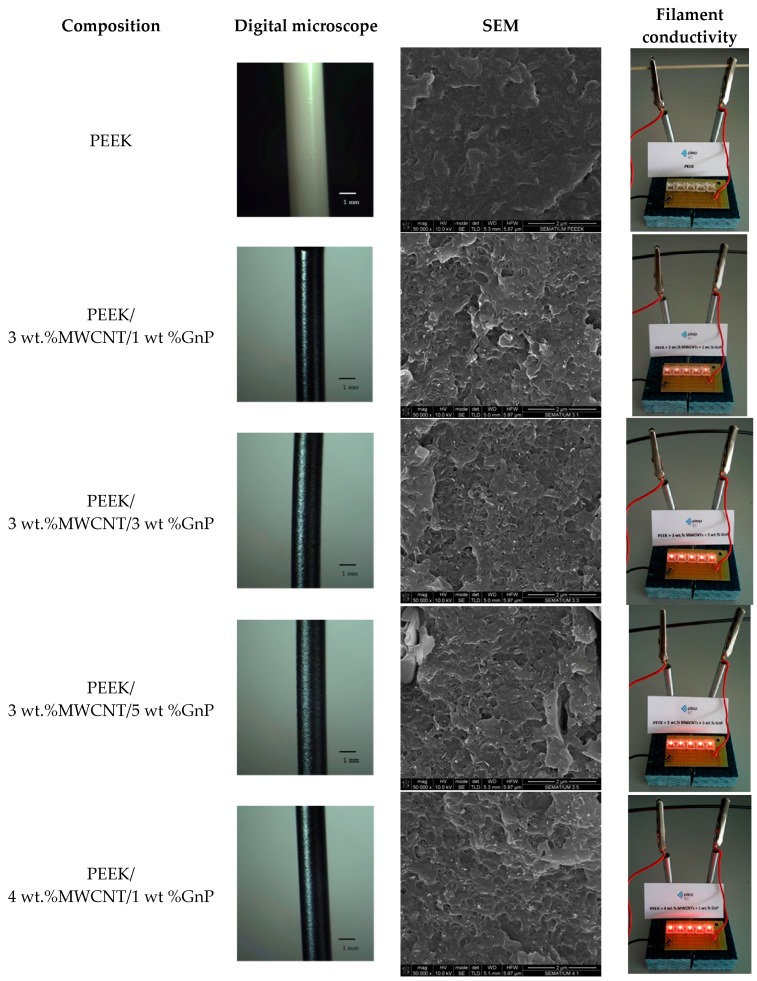

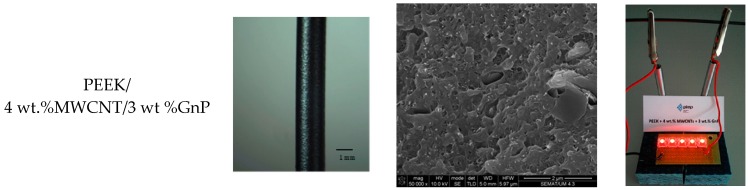
Digital microscope images of selected samples of PEEK and PEEK/CNT/GnP extruded filaments, SEM micrographs of the composite cross-sections, and illustrations of the electrical conductivity of the filaments.

Figure 7 exhibits images of PEEK and PEEK/CNT/GnP filaments obtained on a digital microscope. The filaments were prepared by melt extrusion using a capillary rheometer. For composites containing 3 wt % CNT, the incorporation of GnP up to 5 wt % did not compromise the surface smoothness or the diameter tolerance, in spite of the overall carbon nanoparticle content reaching 8 wt %. This could result from the well-known lubricating effect of graphite. Filaments produced by twin-screw extrusion were cryo-fractured, and their cross-section was observed by SEM, showing the homogeneous distribution of the fillers, as well as good wetting of the nanoparticles with PEEK. All 1–5 wt % CNT filament compositions were within/above the electrical percolation threshold, as evidenced by the increasing light intensity of the LEDs (subjected to 17 volts) with MWCNT/GnP content.

#### 3.2.2. Tensile Properties

The tensile properties of the filaments are presented in Table 3, which shows that the addition of the fillers moderately improved the Young’s modulus and the yield stress, while reducing the ductility. The Young’s modulus and yield stress measured were somewhat lower than the values revealed by the manufacturer (1.48 GPa and 85 MPa vs. 4 GPa and 98 MPa [45]), whilst the elongation at break obtained (>400%) was much higher than the value declared of 45%. These discrepancies could be due to differences in the sample preparation technique, i.e., the extruded filaments tested here should have a much smaller degree of molecular orientation than the injection-molded testing bars used by the manufacturer. As for the filaments produced from the nanocomposites, the ultimate tensile stress (UTS) of approximately 90 MPa was not far from ~100–110 MPa for the values of the same PEEK-containing filaments [22].

#### 3.2.3. Electrical Conductivity

**Figure 8:**
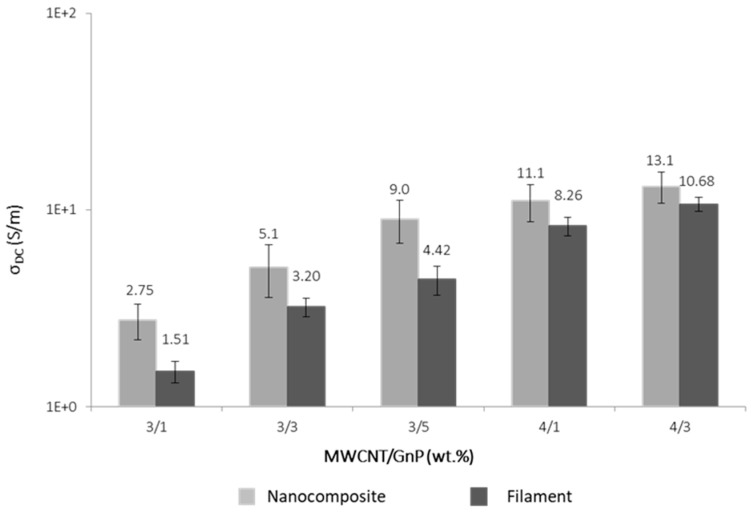
Effect of composition on the DC electrical conductivity of PEEK/CNT/GnP filaments extruded by capillary rheometry, and a comparison with the conductivity of the corresponding nanocomposites.

Figure 8 compares the DC electrical conductivity of the filaments with that of the corresponding nanocomposites. The expected descent in conductivity due to the filament extrusion stage was observed; however, at sufficiently high filler contents, the required electrical conductivity near 10 S/m was still observed.

#### 3.2.4. Differential Scanning Calorimetry

**Figure 9:**
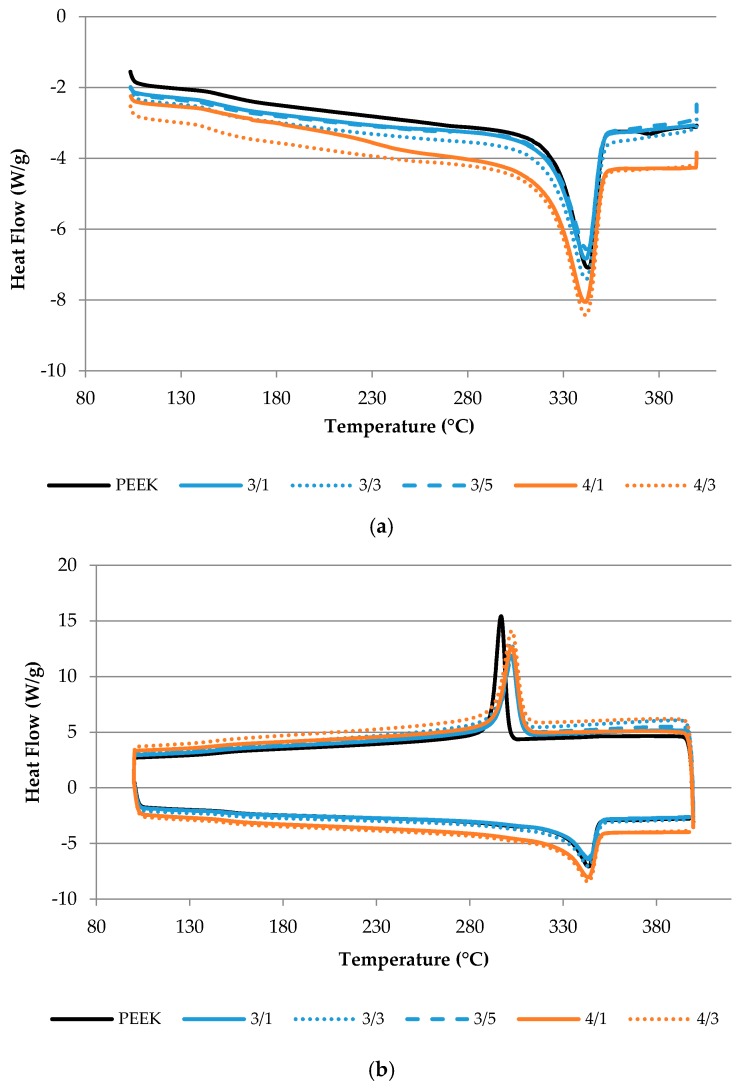
Differential scanning calorimetry (DSC) curves for PEEK and PEEK nanocomposite filaments: (**a**) first heating, and (**b**) cooling and second heating.

Table 4 summarizes the relevant data obtained by DSC analysis of the composites. The melting and crystallization enthalpies were determined after correcting for the composite nanofiller content. Figure 9a illustrates the DSC thermograms obtained for the first heating of PEEK and its composite filaments, while Figure 9b shows the corresponding curves for the cooling and second heating processes. The onset temperature of each peak and the maximum peak temperature were measured, and the enthalpy of each transition was calculated. While the estimate of the peak onset temperature may be affected by considerable uncertainty, the peak temperature can be unequivocally measured. Table 4 shows that the peak temperatures for PEEK and composites did not vary notably from the first to the second heating. The enthalpies associated with the first and second melting processes are also statistically similar. This is an indication that the filaments produced were free from significant residual stress, and that, after melting and cooling, a composite with similar crystallinity degree was obtained. The major difference was observed for the crystallization peak of PEEK that started at a lower temperature compared to its composites, shifting by 7–8 °C, indicating that the polymer crystallization was facilitated in the presence of the nanoparticles. This is a result that is often obtained for polymer nanocomposites.

#### 3.2.5. Thermal Conductivity

**Figure 10:**
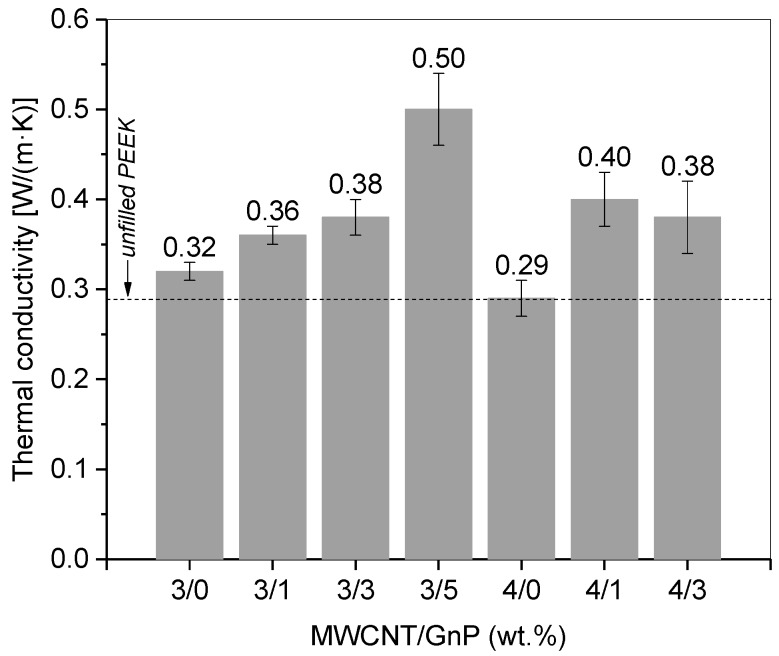
Effect of composition on the thermal conductivity of PEEK/CNT/GnP composites.

The thermal conductivity of the filaments was assessed after re-melting them into discs, as required by the laser flash measurement technique. It was shown before (Figure 6b) that re-melting induced a small loss of electrical conductivity, meaning that nanoparticle re-agglomeration may take place. The discs were prepared by compression molding, i.e., by melting under quasi-quiescent conditions, which may induce a small effect upon the state of dispersion, as well as loss of possible orientation effects of the fillers in the composites. The PEEK grade used in this work presented a thermal conductivity of 0.29 W/(m·K) as reported by the supplier. Filling PEEK with the carbon nanoparticles slightly enhanced the thermal conductivity, as shown in Figure 10. Increasing GnP content further enhanced the thermal conductivity achieved for the composites with MWCNTs only. The highest value of 0.5 W/(m·K) was achieved for the composite containing the higher GnP content, filled with 3 wt % MWCNT and 5 wt % GnP. Composites with 3 or 4 wt % MWCNT containing 1 and 3 wt % GnP showed values between 0.36 and 0.40 W/(m·K).

#### 3.2.6. Thermoelectric Effects

**Figure 11:**
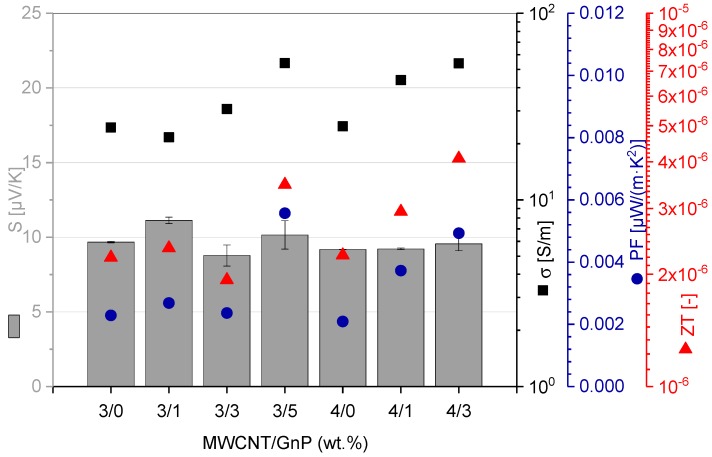
Thermoelectrical properties of PEEK/MWCNT/GnP composites: Seebeck coefficient (*S*), electrical conductivity (σ), power factor (PF), and figure of merit (ZT).

Thermoelectric properties of the composites are interesting in the context of their possible application for the transformation of thermal energy, originating from temperature differences resulting from waste heat, into electrical energy. The PEEK nanocomposites’ thermoelectric properties were investigated on the compression-molded plaques obtained from the filaments, and the characteristic values are presented in Figure 11.

The Seebeck coefficient showed values in the range of 9–11 µV/K. Therefrom calculated PF values ranged between 0.002 and 0.006 µW/(m·K^2^), and the highest ZT value of 4 × 10^−6^ was calculated for the PEEK composite filled with 4 wt % MWCNT and 3 wt % GnP. The Seebeck coefficient values were in the same range as those reported for cellulose/MWCNT (Nanocyl NC3150 grade) films (2–10 wt % MWCNT) [35]. Composites based on polycarbonate-containing 2.5 wt % MWCNTs (Nanocyl NC3150) presented an *S* of 7.5 ± 1 µV/K, resulting in a PF of around 4 × 10^−7^ µW/(m·K^2^) [46]. The ZT was one order of magnitude lower compared to the PEEK composites under investigation here. Sun et al. [46] reported an S of 9.5 µV/K for polyvinylidene fluoride (PVDF)-based composites with 5 wt % MWCNT, and of 12 µV/K at 8 wt % loading (NC7000 grade). A study by Antar et al. [47] reported a maximum *S* of 9 µV/K for composites based on PLA, containing 20 vol % MWCNTs (NC7000), and the reported *ZT* value of 7 × 10^−5^ was higher compared to PEEK composites with much lower loading. Thus, compared to the state of the art, the PEEK-based composites prepared in this work present similar or higher thermoelectric parameters.

#### 3.2.7. Tribological Properties

**Figure 12:**
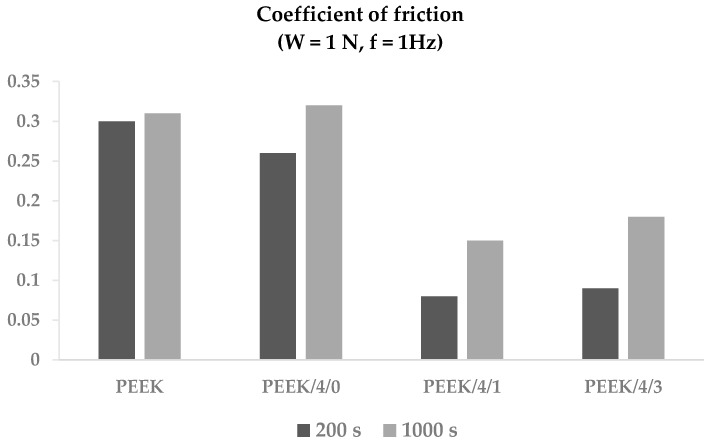
Coefficient of friction of PEEK and PEEK/MWCNT/GnP filaments (values measured at 200 s and 1000 s).

The evolution of the coefficient of friction (*f*_c_) of the filaments against a steel ball was measured in a tribometer during 1200 s (plots presented in Supplementary Materials Figure S1). Figure 12 displays the values measured at 200 and 1000 s. The addition of 4 wt % carbon nanotubes did not significantly alter the value of the coefficient of friction of PEEK, which was approximately 0.3. However, the incorporation of GnP dramatically reduced the value of f_c_ at short times, and, even at longer times, *f*_c_ was half that of the polymer. This behavior was assigned to the lubricating effect of graphite.

### 3.3. 3D-Printed Parts

The five PEEK/MWCNT/GnP filament compositions selected for filament production were 3D printed into the shape of ASTM tensile testing dumbbell bars. All the filaments were reportedly amenable to printing, i.e., easily fed into the machine, having adequate melt viscosity and resistance with good layer adhesion. An example of a relatively complex part manufactured with PEEK and with one of the PEEK nanocomposites—a gear mechanism—can be seen in Reference [34].

**Figure 13:**
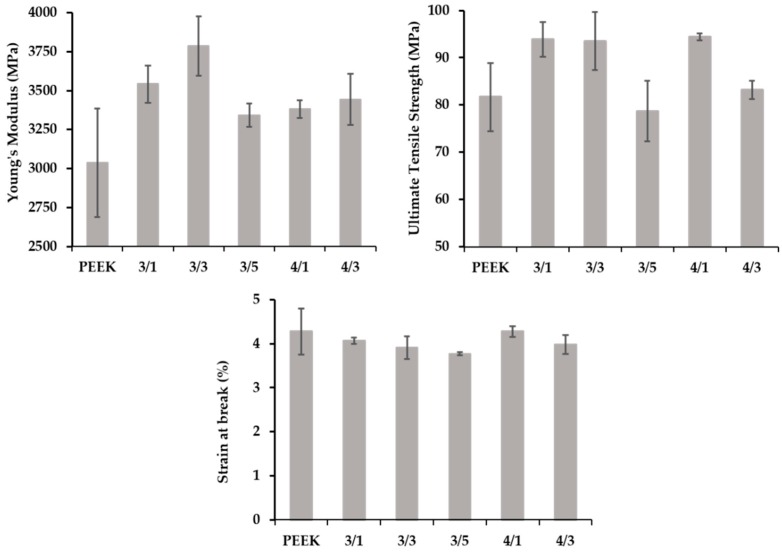
Mechanical properties of 3D-printed tensile bars using PEEK and PEEK/CNT/GnP filaments.

**Figure 14:**
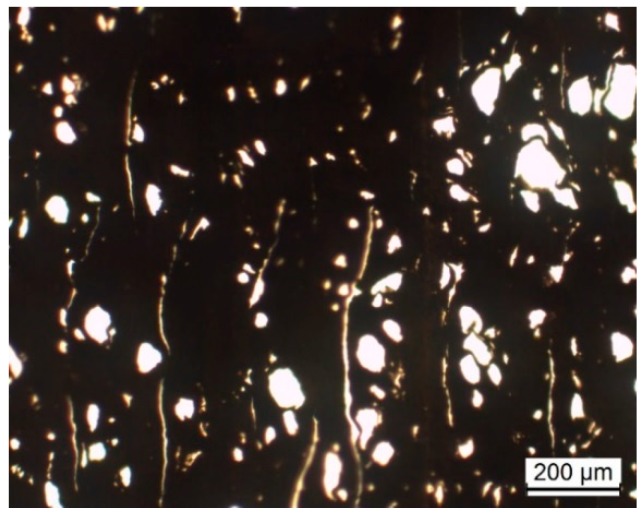
Optical microscopy of the cross-section of the 3D-printed dumbbell sample.

Figure 13 displays the Young’s modulus, ultimate tensile strength, and elongation at break of the various 3D-printed tensile bars. The values were relatively uniform, with the composites consistently presenting a fairly higher modulus. Compared to the extruded filaments (Table 3), the 3D-printed parts showed an improved modulus and a higher UTS, but a much smaller elongation at break. The porosity of the printed parts, as observed by optical microscopy and depicted in Figure 14, could govern this behavior. Moreover, these results contrast with the measurements of Berreta et al. [22] for FDM filaments produced from the same PEEK and with 1 and 5 wt % CNT, where a significant decay in UTS was reported for the 3D-printed bars relative to the respective filaments. For example, in the present work, a printed part made of PEEK/4 wt % MWCNT/1 wt % GnP presented a UTS of 92 MPa, which contrasts with 55 MPa for PEEK/5% CNT measured by those authors.

**Figure 15:**
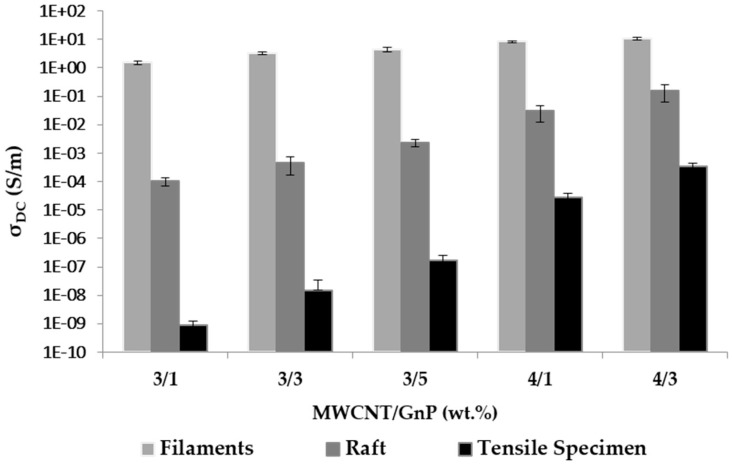
DC electrical conductivity for PEEK/CNT/GnP filaments, (contour of first row during 3D printing) and 3D-printed part using fused deposition modeling (FDM) (ASTM tensile specimen).

Figure 15 shows the electrical conductivity of the extruded filaments, rafts, and 3D tensile specimens. The raft (also known as brim) is automatically generated by the printer and consists of additional material around the contour of the part in the bottom row, with the aim of improving the adhesion with the support and stabilizing the printing sequence. The data for the filaments were the same as that in Figure 8. The progressive reduction in the mechanical properties for a filament, first row, and FDM part reported by Berreta et al. [22] was perceived here for the conductivity. Even in the case of the higher-filled materials, a loss of conductivity of two orders of magnitude was observed between filament, raft, and specimen. This observation may partially result from the melting of the conductive filament under quasi-quiescent conditions that allowed partial re-agglomeration of the carbon nanoparticles. Another relevant factor derived from the limitations in separating the surface and volumetric contributions to the electrical conductivity. While the filament had a relatively small surface compared to its cross-section, the printed specimen was made of filament elements separated by gaps/pores. Consequently, in this case, the surface-to-cross-section ratio was quite different. Therefore, an interesting line of research would be to investigate the deposition strategy during 3D printing, in order to maximize electrical conductivity.

### 3.4. Scale up of the Filament Production

**Figure 16:**
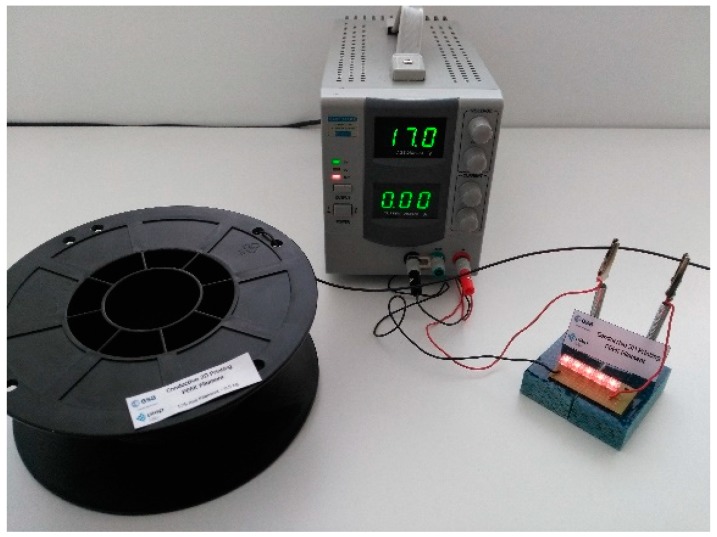
Spool with electrically conductive PEEK nanocomposite filament.

Finally, nanocomposite filaments with 4 wt % CNT and 3 wt % GnP were produced at kilogram scale, presenting properties comparable to the research-scale filaments described above. The filaments were wound into spools, each containing 0.5 kg of filament. The electrical conductivity and filament diameter were measured statistically, and the average values obtained were 7 ± 2 S/m and 1.74 ± 0.03 mm, respectively. Thus, the possibility of scaling up the nanocomposite filament production to the kilogram scale by extrusion processing, maintaining the filament dimensions and electrical conductivity at the required level, was demonstrated. Figure 16 depicts a filament spool and illustrates its electrical conductivity. The major filament characteristics are reported in the technical datasheet provided in the Supplementary Materials.

## 4. Conclusions

The objective of the present work was to develop electrically conductive PEEK-based filaments, with good mechanical properties, suitable for 3D printing by fused deposition modeling. Both the manufacture of the nanocomposites and the extrusion into filaments were performed using scalable methods toward industrial production.

PEEK/MWCNT nanocomposites revealed an electrical percolation threshold taking place between 2 and 3 wt % CNT. The incorporation of GnP induced a further increase in the electrical conductivity levels attained, albeit moderate. Combinations of 3 wt % CNT with higher loads of GnP, or of 4 wt % CNT with lower loads of GnP, showed consistent electrical conductivities of approximately 10 S/m. Interestingly, the incorporation of GnP into the matrix had a less adverse effect on the processability than that of MWCNT, as showed by the minor changes caused in the melt flow index, in the motor torque of the extruder, and in the melt temperature.

The addition of MWCNT/GnP to PEEK reasonably improved the Young’s modulus and the yield strength, while reducing the ductility of PEEK filaments. The DSC data indicated that the filaments produced were free from significant residual stress, and that, after melting and cooling, composites with similar crystallinity degree were obtained, even if polymer crystallization was facilitated in the presence of the nanoparticles. Although the electrical conductivity of the filaments was lower than that of the equivalent nanocomposites, at sufficiently high filler contents, values near to 10 S/m were still observed. The thermal conductivity was also enhanced. Moreover, the thermoelectric parameters were similar to or higher than those reported in the literature for other PEEK-based composites. In this case, the lubricating effect of graphite seen for the nanocomposites caused a noteworthy reduction in the friction coefficient. This behavior should be relevant for applications requiring tribological properties, such as gears (see an example in Reference [34]).

The 3D-printed tensile bars showed an improved modulus and a higher UTS, but a much smaller elongation at break as compared to the extruded filaments. The usual porosity of parts produced by FDM could influence this response. A loss of electrical conductivity of two orders of magnitude was observed from filament to raft and to 3D-printed part. Again, the porosity of the 3D-printed parts should affect the surface and volumetric contributions to the transport properties. Therefore, an interesting line of research would be to investigate the effect of the 3D-printing processing parameters on the electrical conductivity and other physical and mechanical properties.

## Figures and Tables

**Table 1:** Properties of commercial grades of multi-walled carbon nanotubes (MWCNT) and graphene nanoplates (GnP), according to the suppliers.

Nanoparticle/Manufacturer	Length/Width (μm)	Diameter/Thickness (nm)	Surface Area (m^2^/g)	Carbon Purity (%)	Bulk Density (g/cm^3^)
MWCNT NC 7000/Nanocyl, Belgium	1.5	9.5	250–300	>90	0.066
xGnP-M/XG Science Inc, USA	15	6–8	120–150	>99.5	0.03–0.10

**Table 2:** Effect of CNT and GnP content on compounding parameters.

Compositiong		Torque (%)	Pressure (Bar)	Melt Temperature (°C)
Polyetheretherketone (PEEK)		30–32	21–24	368
**MWCNT (wt %)/GnP (wt %)**	1/0	35	25	369
	2/0	35	28	372
	3/0	38	28	376
	4/0	40	32	379
	6/0	42	37	386
	3/1	37–39	25–28	376
	3/2	36–40	23–28	378
	3/3	36–40	23–30	379
	3/4	36–39	26–32	379
	3/5	37–38	28–30	380
	3/6	37–39	29–32	381

**Table 3:** Mechanical properties of PEEK and PEEK/CNT/GnP filaments.

Composition	*E* (GPa)	σ_y_ (MPa)	UTS (MPa)	ε_break_ (%)
PEEK	1.48 ± 0.1	85± 2	-	>400
PEEK/3/1	1.9 ± 0.1	84 ± 1	78.6 ± 0.7	42 ± 3
PEEK/3/3	1.7 ± 0.1	88 ± 3	82 ± 4	39 ± 5
PEEK/3/5	1.8 ± 0.1	92 ± 1	89 ± 1	27 ± 7
PEEK/4/1	1.6 ± 0.1	90 ± 3	86 ± 3	53 ± 4
PEEK/4/3	1.60 ± 0.1	92 ± 1	88 ± 1	53 ± 8

**Table 4:** PEEK and nanocomposite differential scanning calorimetry (DSC) crystallization and melting data.

Material		First Heating		Second Heating		Cooling	
		*T*_m_ (°C)	∆*H* (J/g)	*T*_m_ (°C)	∆*H* (J/g)	*T*_m_ (°C)	∆*H* (J/g)
PEEK		342.9 ± 0.2	36.0 ± 2	343.4 ± 0.2	35.0 ± 1.0	296.9 ± 0.2	40.0 ± 2.0
MWCNT/GnP Ratio	3/1	341.9 ± 0.03	37.0 ± 0.5	343.8 ± 0.1	37.8 ± 0.1	302.1 ± 0.1	36.3 ± 0.1
	3/3	342.2 ± 0.1	37.3 ± 0.1	343.6 ± 0.1	39.0 ± 1.0	302.1 ± 0.2	36.0 ± 1.0
	3/5	343.0 ± 1.0	38.0 ± 1.0	343.5 ± 0.2	38.5 ± 0.7	302.4 ± 0.1	36.4 ± 0.4
	4/1	341.6 ± 0.1	40.0 ± 1.0	343.4 ± 0.04	41.1 ± 0.3	302.4 ± 0.1	39.2 ± 0.5
	4/3	341.9 ± 0.1	39.0 ± 1.0	343.2 ± 0.3	40.9 ± 0.9	302.5 ± 0.2	39.0 ± 1.0

## Supplementary Materials

The following are available online at http://www.mdpi.com/2073-4360/10/8/925/s1.
