# Supplementary material for: Electrically Conductive Polyetheretherketone Nanocomposite Filaments: From Production to Fused Deposition Modeling
Source: Polymers (Basel). 2018 Aug 18;10(8):925. doi: 10.3390/polym10080925 (PMC6403709; doi:10.3390/polym10080925)
Supplement: Supplementary file 1 [file polymers-10-00925-s001.pdf]

Supplementary

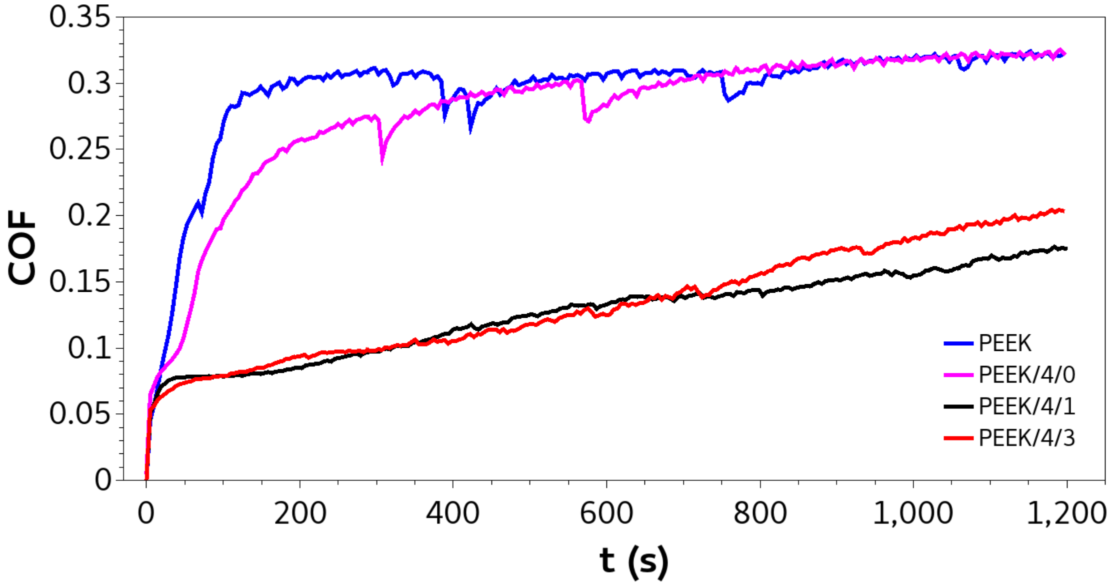

Figure S1

Conductive 3D Printing PEEK Filament

Physical Properties	Value	Unit	Standard
Specific gravity	1,31	g/cm³	ISO 1183
Melt flow rate (360 °C / 10 kg)	2	g/10 min	ASTM D1238

Mechanical Properties	Value	Unit	Standard
Tensile Modulus	1,60 ± 0,07	GPa	ASTM D2256
Tensile Strength	92 ± 1	MPa	
Tensile Elongation at Break	53 ± 8	%	

Thermal Properties	Value	Unit	Standard
Melting Temperature	343	°C	ISO 11357

Electrical Properties	Value	Unit	Standard
Volume Conductivity (23 °C)	7	S/m	Internal test method
Loss Tangent (23 °C / 1 MHz)	2,5	n/a	
Dielectric Constant (23 °C / 1 kHz)	10^6	n/a	

	Value	Unit
Diameter	1,75	mm

PIEP Innovation in Polymer Engineering
Universidade do Minho, Campus de Azurém
4800-058 Guimarães - Portugal
t.: (+351) 253 510 050; f.: (+351) 253 510 059
geral@piep.pt
www.piep.pt

January 2017
